# Occlusal Force Evaluation in Growing Patients With Posterior Crossbite: A Case‐Control Study

**DOI:** 10.1155/ijod/3510369

**Published:** 2025-12-11

**Authors:** Mauro Lorusso, Michele Tepedino, Carlotta Fanelli, Rosa Esposito, Donatella Ferrara, Fariba Esperouz, Lucio Lo Russo, Domenico Ciavarella

**Affiliations:** ^1^ Department of Clinical and Experimental Medicine, Dental School of Foggia, University of Foggia, Foggia, Italy, unifg.it; ^2^ Department of Biotechnological and Applied Clinical Sciences, Dental School of L’Aquila, University of L’Aquila, L’Aquila, Italy, univaq.it

**Keywords:** dynamometer, force discrepancies, occlusal force, posterior crossbite

## Abstract

**Objective:**

The study aimed to assess occlusal force in patients with posterior crossbite using a dynamometer. Additionally, the discrepancies in force between the crossbite side and the opposite side of the arches were evaluated.

**Methods:**

The occlusal force of 52 patients with posterior crossbite was measured and compared with that of 52 patients with normal occlusion. The Wilcoxon signed‐rank test was performed to compare the force of each side. Because the data had a non‐normal distribution, to compare the total occlusal force of the two groups, the Mann–Whitney test was performed. Finally, to compare the force between the two sides, using a symmetry index determined by the difference in force between the sides, the Mann–Whitney test was used.

**Results:**

In the group of patients with crossbite, the crossbite side showed greater occlusal force than the opposite side. The total occlusal force was lower in the crossbite group than in the control group.

**Conclusions:**

In patients with posterior crossbite, an alteration in occlusal force, characterised by a higher value on the crossbite side than on the opposite side, was observed. In addition, these patients showed lower total occlusal force compared to the control group.

## 1. Background

Posterior crossbite is a frequent malocclusion due to a transverse discrepancy between the maxilla and mandible, and it occurs most frequently in deciduous and mixed dentitions, with prevalence rates ranging from 7.5% to 22% [1]. It can develop in deciduous or early mixed dentition and may have a skeletal or dental aetiology [2]. In addition, the aetiology may be functional or a combination of multiple factors [3]. These factors include, in addition to heredity, crowding, premature loss or retention of deciduous teeth, palatal cleft sucking habits and altered nasal breathing caused, for example, by enlarged tonsils and adenoids [4]. Unilateral posterior crossbite with functional displacement of the mandible to the side of the crossbite is the most common form of posterior crossbite, known as functional crossbite [5]. This condition occurs as a result of an altered transverse dimension of the maxillary arch, and its prevalence ranged from 80% to 97% of posterior crossbites in mixed dentition [6]. Some authors suggest that posterior crossbite may increase the risk of subsequent temporomandibular joint disorders [7] because the condyle can have an asymmetrical position, with the crossbite side centred in the fossa and the opposite side displaced lower and forward in the fossa [8]. Pinto et al. [9] suggested that early treatment of posterior crossbite is crucial to avoid long‐term effects on normal jaw and tooth growth. Crossbite treatment induces favourable changes in jaw kinetics and normalises asymmetrical functional aberrations and stomatognathic muscle activity [10]. Grassia et al. [11] observed that palatal expansion results in favourable changes in the intermolar and intercanine distances of both arches. In addition, the effects of jaw expansion on airway and cervical posture have been demonstrated, as an increase in airway patency results in a favourable change in cervical posture [12]. Indeed, failure in the treatment of posterior crossbite can cause alterations in some masticatory muscles, such as the masseter and temporalis muscles in children, and may promote the development of craniomandibular disorders in adolescents [13]. Therefore, early treatment of crossbite is commonly recommended to reduce the risk of developing skeletal asymmetries resulting from abnormalities in chewing function between the right and left sides. Children with a unilateral posterior crossbite exhibit several types of unusual chewing patterns when chewing on the affected side, which were first documented by Ahlgren [14] in 1967. The significant presence of reverse sequence chewing cycles, due to jaw movement during the closing phase of mastication, was observed during chewing on the side of the crossbite in patients with posterior crossbite [15]. Reverse chewing cycles show an abnormal and restricted kinematic pattern in the frontal plane, characterised by limited lateral displacement of the mandible compared to the pattern on the unaffected side, which shows physiological morphology [16]. This results in severe mastication asymmetry. It is evident that in the crossbite, there is an altered pattern of muscle activation due to non‐physiological mandibular kinetics, which, if untreated, can facilitate the development of functional and skeletal asymmetries. The resulting facial asymmetry is due to the adaptation of hard and soft tissues, resulting in greater development of the non‐crossbite side and underdevelopment of the cross side. Whereas to date there are numerous studies regarding impaired muscle activity in patients with crossbite, studies regarding muscle force in these patients are limited. Analysing occlusal force in these patients is very important, especially to understand how the difference in force between the sides can be managed and evaluated during therapy.

Clinically, assessing occlusal force in growing patients with posterior crossbite is crucial, as muscular asymmetries may lead to skeletal alterations in adulthood. Proper function of the masticatory muscles and maintenance of muscular balance are essential, since any deviations can impact growth and affect normal masticatory physiology. This issue requires careful consideration, as muscular alterations in growing patients can be effectively managed through appropriate functional therapy. However, if left unaddressed, these alterations may result in growth anomalies that can cause irreversible skeletal asymmetries. These asymmetries cannot be addressed solely through orthodontic therapy and often require a surgical approach for correction. Moreover, an asymmetrically functioning system imposes uneven loading on the temporomandibular joint, potentially leading to joint disorders that limit masticatory function and cause pain.

The aim of the present study was to analyse total and per‐side occlusal force in patients with posterior crossbite and compare them with a control group of patients without transverse discrepancy using the Innobyte. The null hypothesis was that there are no differences in occlusal force between crossbite patients and the control group.

## 2. Methods

All procedures outlined in this research protocol adhered to the Declaration of Helsinki and received approval from the Ethics Committee of the University of Foggia (Approval number 17/CE/2024). This study adheres to the guidelines outlined in Strengthening the Reporting of Observational Studies in Epidemiology (STROBE) for observational studies [17].

Two groups of patients were selected: the first group included patients with unilateral crossbite who subsequently underwent orthodontic treatment, and the second group served as a control, consisting of patients without crossbite. The inclusion criteria were the following: patients with posterior crossbite and class I malocclusion, symmetric dental formula and a normo‐divergent facial pattern (30.5° ≤ SN‐GOME ≤ 35.5°). Patients with agenesis, periodontal disease, fixed or removable prostheses, loss of one or more teeth, presence of scissor‐byte, unilateral chewing, signs and symptoms of temporomandibular joint disorders, history of trauma, heavily restored teeth in the molar area, previous orthodontic treatment or previous maxillofacial or plastic surgery were excluded from the study. Cephalometric analysis was performed by an experienced orthodontist.

A power analysis (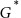
Power 3.1.9.2, Franz Faul, Universitat Kiel, Germany) revealed that to detect an effect size of 0.5 [18] with a Mann–Whitney test, considering an *α* error probability of 0.05 and a power (1 − *β*) of 0.80, 51 subjects for each group would be needed. Therefore, 104 Caucasian patients (54 males and 50 females) with a mean age of 9.2 ± 0.5 were enrolled in the study. The study sample included two groups: 52 patients with posterior crossbite (mean age: 9 ± 0.45) and a control group of 52 patients (mean age: 9.4 ± 0.48).

### 2.1. Measuring Device

The Innobyte medical device is a dynamometer specifically designed to measure the maximum occlusal force in the total or unilateral dental arch. The Innobyte system consists of two components: the main device and the mouthpiece. The device can measure occlusal force in the range of 0–2000 N with an accuracy of 5%. The mouthpiece contains a fluid, and when force is applied to the mouthpiece, it transmits the applied force throughout the medium, which is then read by the sensors. The main device has an LED display on which the force, expressed in Newtons, is shown. With each recorded measurement, the display shows both the total force and the forces on the left and right sides [19].

### 2.2. Study Procedure

**Figure 1:**
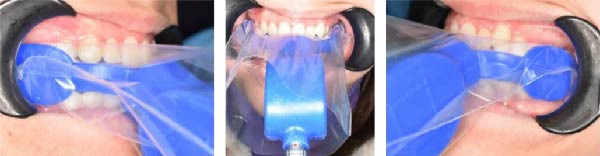
Occlusal force measurement with the Innobyte.

The study protocol was explained to patients, and patients’ parents signed informed consent to participate in the study. The patients were seated in the dental chair with the head positioned normal to the body, and the mandible parallel to the floor. The recording sensor was inserted intraorally between the dental arches, positioning the central notch between the patient’s central incisors. Recording began by pressing the button on the handlebar, and the patient was instructed to occlude firmly in a maximal voluntary clench. After the force measurement, the mouthpiece was removed, and the patient was asked to swallow. Following the swallowing act, the patient remained in the rest position for 10 s, after which the next measurement was taken. A multibite scan, consisting of three recorded bites, was performed for each participant to minimise error [19]. Figure 1 shows the measurement of occlusal force using the Innobyte.

### 2.3. Determination of Preferred Chewing Side

The preferred chewing side was determined using a modified visual spot‐checking method [20, 21]. Participants were asked to perform natural chewing of sugar‐free chewing gum over the posterior teeth. After 15 s, participants were asked to stop chewing and smile to show the position of the gum, and its presence on the left or right side was noted. The procedure was repeated seven times, successively, at 5‐s intervals. Participants with a preferred chewing side were those that revealed the gum on the same side a minimum of five times. These patients were excluded from the study.

### 2.4. Statistical Analysis

Data were analysed using GraphPad Prism software 6.0 (GraphPad Prism Software, San Diego, CA, USA). Since the data had a non‐normal distribution, the Wilcoxon signed‐rank test was performed to compare the force between the sides of the groups. To compare the total occlusal force of the two groups, the Mann–Whitney test was performed, as the data had a non‐normal distribution. To compare the force on the two sides, an index of symmetry (∆) was calculated by determining the force difference between the left and right hemiarches in the two groups using the formula ∆ = force on the cross side—force on the opposite side, while in the control group it was calculated as ∆ = force on the left side—force on the right side, followed by the Mann–Whitney test, as the data had a non‐normal distribution. Statistical significance was set as *p*  < 0.05.

## 3. Results

The average occlusal force values according to malocclusion class and side are reported in Table 1. Also, Table 2 summarises the comparison of occlusal forces between the right and left sides. In the crossbite group, a statistically significant difference was observed between sides (*p*  < 0.001), with the crossbite side exhibiting greater occlusal force than the contralateral side. Conversely, in the control group, no significant inter‐side difference was found (*p* = 0.101). The comparison of total occlusal force between the two groups, presented in Table 3, revealed a statistically significant difference (*p*  < 0.001). Specifically, the crossbite group demonstrated a lower total occlusal force compared with the control group. Finally, the difference in occlusal force between sides within groups, shown in Table 4, was also statistically significant (*p*  < 0.001). Therefore, based on the results, the null hypothesis was rejected.

**Table 1:** Force values in groups categorised by malocclusion class and side.

Cohort	Total force
Crossbite	387.15 ± 131.95
Control group	601.39 ± 212.52
Crossbite—crossbite side	223.79 ± 87.78
Crossbite—opposite side	167.78 ± 61.58
Control group—right side	307.07 ± 138.25
Control group—left side	286.91 ± 109.33

*Note:* Force values are expressed in Newtons (N). Data are presented as mean ± standard deviation (SD).

**Table 2:** Wilcoxon signed‐rank test: force comparison between the two sides.

Crossbite group	Crossbite side	Opposite side	*p*
	223.79 ± 87.78	167.78 ± 61.58	<0.001
Control group	Right side	Left side	—
	307.07 ± 138.25	286.91 ± 109.33	0.101

*Note:* Force values are expressed in Newtons (N). Data are presented as mean ± standard deviation (SD).

**Table 3:** Mann–Whitney test: comparison of total force between the groups.

Total force	Crossbite group	Control group	*p*
	387.15 ± 98.99	601.39 ± 149.19	<0.001

*Note:* Force values are expressed in Newtons (N). Data are presented as mean ± standard deviation (SD).

**Table 4:** Mann–Whitney test for the force difference between the left and right side in the groups.

Force difference ∆ = left–right or crossbite side‐opposite side	Crossbite group	Control group	*p*
	56.01 ± 17.08	−20.16 ± 6.76	<0.001

*Note:* Force values are expressed in Newtons (N). Data are presented as mean ± standard deviation (SD).

## 4. Discussion

Crossbite is a discrepancy in the transverse plane that is very complex to manage due to the associated functional and muscular changes [22]. Therefore, orthodontic treatment aims to achieve dental, skeletal and muscular rehabilitation [23]. Muscles provide support to the skeletal components, enabling joint movements and providing masticatory function in conjunction with the teeth. The aim of the present study was to analyse occlusal force in patients with unilateral crossbite and compare it with a control group of patients with Class I malocclusion. Occlusal force is correlated with several factors such as age, gender, periodontal status and temporomandibular disorders [24, 25]. This is the first study to use Innobyte as a system for measuring occlusal force in patients with crossbite malocclusion. The Innobyte was chosen to assess occlusal force because of its ease of use and its ability to detect total force and per‐side force by showing values in Newtons on the LED handpiece. When comparing the force between the two sides, a significant difference was revealed in the crossbite group of patients. In this group, the crossbite side showed greater force than the opposite side (223.79 vs. 167.78 N). In contrast, Sonnesen et al. [26] found no significant difference between the sides in children with posterior crossbite. In the control group, no significant differences were observed between the two sides, although one side had a slightly greater force than the opposite. In a prospective study, Sonnesen and Bekke [27] observed that occlusal force during orthodontic treatment was significantly lower on the crossbite side than on the contralateral side. The authors also noted that the force decreased after treatment and increased during the retention phase. This change in force could be explained by occlusal and periodontal mechanoreceptor adaptations in association with muscle changes during the phases of orthodontic treatment.

Regarding total force, a significant difference was found between the two groups. In fact, total force was lower in the crossbite patient group than in the control group. In accordance with the results of the present study, Castelo et al. [28] observed that total force was lower in the group of patients with crossbite than in the group with normal occlusion. In contrast, Rentes et al. [29] did not observe significant differences in force between the crossbite group and the control group. According to the results of the present study, Sonnesen et al. [26] reported lower occlusal force in a group of patients with crossbite compared with the control group. Moreover, they observed that the difference in force did not decrease with age and development. This difference in force between the groups may be explained by the lower occlusal contacts in crossbite patients compared with the control group. However, the role of the muscles must also be considered since, as shown by previous studies [30, 31], one of the factors that determines maximum occlusal force is the size of the masseter muscle. Kiliaridis et al. [32] observed that the masseter muscle was thinner on the crossbite side than on the opposite side. As observed by Alarcón et al. [33], unilateral posterior crossbite without functional mandibular lateral shift was associated with lower electromyographic activity of the masseter muscle on the crossbite side during maximal clenching in maximal intercuspation, compared to children with normal occlusion. In a review of the literature, Iodice et al. [34] showed that the electromyographic activity of the masticatory muscles may be different on the crossbite side than on the opposite side. Therefore, it is possible to hypothesise that the lower force observed in the present study in crossbite patients compared with the control group is the result of structural and functional asymmetry of masticatory muscles such as the masseter. This functional asymmetry must be carefully evaluated, as overtime, altered function could result in asymmetric skeletal growth.

Orthodontic therapy is essential for the correction of posterior crossbite, as it aims to re‐establish proper occlusal relationships, enhance masticatory function and prevent long‐term skeletal asymmetries. Early interceptive interventions, including maxillary expansion or the use of functional appliances, have been demonstrated to optimise occlusal force distribution, increase the number of teeth in occlusal contact, and mitigate the risk of temporomandibular joint disorders. Al‐Khateeb et al. [35] reported that children with unilateral posterior crossbite exhibited significantly lower maximum bite force and fewer occlusal contacts compared to healthy controls. These results are consistent with the observations of the present study and those reported by Castelo et al. [28] However, it is important to consider the significant force asymmetry between the crossbite side and the contralateral side. Muscular asymmetries in the masticatory and functional occlusal system must be managed correctly and early in growing patients, as these alterations can develop into skeletal asymmetries that cannot be resolved with orthodontic therapy. In adult subjects, such imbalances may lead to significant joint dysfunction due to unbalanced loading on the two sides. Moreover, the periodontium can be compromised by prolonged asymmetric occlusal loads. For these reasons, an early therapeutic approach with appropriate functional therapy is essential.

To compare the force between the left and right sides within the groups, a symmetry index was calculated. This index represents the difference in force between the left and right sides. In the case of crossbite, the difference between the crossbite side and the opposite side was calculated. The comparison of force differences between the two groups was statistically significant. In the crossbite patient group, the difference in force between the two sides was greater than in the control group. Specifically, as previously described, the crossbite side had statistically significantly greater force than the opposite side. In contrast, in the control group, the difference in force between the two sides was smaller. As shown in Table 3, the comparison between the two sides was not statistically significant. The difference in force between the two sides in patients with crossbite was not due to unilateral chewing because using the visual spot‐checking method, patients with a preferred chewing side were excluded from the study.

The results of the present study have important clinical implications. In patients with a crossbite, there is a difference in force between the two sides that needs careful evaluation, especially during orthodontic treatment. Understanding the complex dynamics of occlusal force is crucial for improving therapies and planning more effective and predictable orthodontic treatments.

### 4.1. Limitations of the Study

Since the patients analysed were not randomly recruited from the population but were selected from a clinic, some inherent bias may be present. Future investigations should include a larger sample size and evaluate additional factors that may influence occlusal force, exploring their effects on malocclusion and orthodontic treatment.

## 5. Conclusion

The results of the present study showed important occlusal force characteristics in patients with crossbite. Specifically, it was observed that these patients exhibited lower occlusal force compared to the control group. Additionally, it was highlighted that the force was greater on the crossbite side than on the opposite side. These observations are of fundamental importance and partly explain the difficulty of orthodontic treatment and associated recurrence. In these patients, orthodontic therapy should not only correct transverse discrepancies but also rebalance muscle activity and thus neuromuscular function.

## Disclosure

All authors read and approved the final version of the manuscript.

## Conflicts of Interest

The authors declare no conflicts of interest.

## Author Contributions

Conceptualisation, writing – original draft preparation, and project administration: Domenico Ciavarella. Methodology, writing – review and editing, and project administration: Mauro Lorusso. Validation: Rosa Esposito. Formal Analysis: Donatella Ferrara and Lucio Lo Russo. Investigation: Carlotta Fanelli. Data Curation: Michele Tepedino. Visualisation: Fariba Esperouz. Supervision: Lucio Lo Russo.

## Funding

The authors declare that they have not received funding.

## Data Availability

The data that support the findings of this study are available from the corresponding author upon reasonable request.
